# Methods for Identifying Epilepsy Surgery Targets Using Invasive EEG: A Systematic Review

**DOI:** 10.3390/biomedicines12112597

**Published:** 2024-11-13

**Authors:** Karla Ivankovic, Alessandro Principe, Riccardo Zucca, Mara Dierssen, Rodrigo Rocamora

**Affiliations:** 1 Department of Medicine and Life Sciences, Universitat Pompeu Fabra (UPF), 08003 Barcelona, Spain; kivankovic@researchmar.net (K.I.);; 2 Hospital del Mar Research Institute, 08003 Barcelona, Spain; 3 Donders Institute for Brain, Cognition and Behaviour, Radboud University, 6525 GD Nijmegen, The Netherlands; 4 Centre for Genomic Regulation (CRG), The Barcelona Institute for Science and Technology, 08003 Barcelona, Spain; 5 Biomedical Research Networking Center on Rare Diseases (CIBERER), 28029 Madrid, Spain

**Keywords:** pharmacoresistant epilepsy, quantitative EEG, invasive EEG, biomarkers, outcome prediction, epilepsy surgery

## Abstract

Background: The pre-surgical evaluation for drug-resistant epilepsy achieves seizure freedom in only 50–60% of patients. Efforts to identify quantitative intracranial EEG (qEEG) biomarkers of epileptogenicity are needed. This review summarizes and evaluates the design of qEEG studies, discusses barriers to biomarker adoption, and proposes refinements of qEEG study protocols. Methods: We included exploratory and prediction prognostic studies from MEDLINE and Scopus published between 2017 and 2023 that investigated qEEG markers for identifying the epileptogenic network as the surgical target. Cohort parameters, ground truth references, and analytical approaches were extracted. Results: Out of 1789 search results, 128 studies were included. The study designs were highly heterogeneous. Half of the studies included a non-consecutive cohort, with sample sizes ranging from 2 to 166 patients (median of 16). The most common minimum follow-up was one year, and the seizure onset zone was the most common ground truth. Prediction studies were heterogeneous in their analytical approaches, and only 25 studies validated the marker through post-surgical outcome prediction. Outcome prediction performance decreased in larger cohorts. Conversely, longer follow-up periods correlated with higher prediction accuracy, and connectivity-based approaches yielded better predictions. The data and code were available in only 9% of studies. Conclusions: To enhance the validation qEEG markers, we propose standardizing study designs to resemble clinical trials. This includes using a consecutive cohort with long-term follow-up, validating against surgical resection as ground truth, and evaluating markers through post-surgical outcome prediction. These considerations would improve the reliability and clinical adoption of qEEG markers.

## 1. Introduction

The seizures of one-third of epilepsy patients cannot be controlled by antiepileptic drugs. Drug-resistant patients undergo invasive intracranial EEG (iEEG) explorations, where clinical specialists visually inspect the signals to determine the epileptogenic area. Using synthesized anatomical and electrophysiological information, clinicians identify the optimal margins for surgical resection. The goal of epilepsy surgery is to achieve seizure freedom, defined as the absence of seizures or the absence of seizures that have an impact on a patient’s daily life [1]. However, current diagnostics do not reliably predict the surgical targets nor ensure post-surgical seizure freedom, with only about 50–65% of patients achieving long-term seizure freedom [2,3], depending on the epilepsy type.

There is no standard measure of epileptogenicity, and current diagnostics rely on estimates derived from anatomical and electrophysiological observations. The challenge arises from the significant variability among patients, including differences in epilepsy types, duration and semiology, and custom electrode implantation schemes that may lead to undersampling. Additionally, individual biological variability further complicates accurate diagnosis, such as in case of focal cortical dysplasia type I, caused by somatic mutations in genes involved in different signaling pathways, such as MAP2K1 and PTPN11 in the RAS/MAPK pathway [4]. Despite these challenges, common patterns exist, motivating the research community to identify a general fingerprint of epileptogenicity and to develop theoretical frameworks for conceptualizing seizure organization. Historically, Talairach and Bancaud introduced the concept of the “epileptogenic zone” as the origin of seizures visible on iEEG [5]. This concept has evolved into the “epileptogenic network” (EN) model, supported by advancements in brain imaging technology and neuroscience [6,7]. Evidence increasingly supports that seizures arise from a distributed, dynamic network of anatomically and functionally connected neural populations, rather than from an isolated focus. However, a quantitative biomarker to pinpoint the EN and guide epilepsy surgery is not yet available in clinical practice.

Many quantitative iEEG (qEEG) biomarkers have been proposed, including measures of local field potentials (LFPs) or connectivity between neural populations. Additionally, dynamic computational models and specialized deep learning classifiers have also been developed. Methods for identifying the EN are most commonly reported as retrospective prognostic studies that follow a similar study design strategy. Typically, the iEEG channels (electrode pairs) are considered as network nodes, representing a sample of the patient’s brain network. An analytical method is applied to the signals from these nodes to identify the epileptogenic ones. The predicted network is then compared to the ground truth in a retrospective cohort of patients for validation. Such studies can be exploratory, quantifying a biomarker from iEEG and comparing it between epileptogenic and non-epileptogenic brain areas, as well as between patients with good and poor surgical outcomes. Alternatively, they can be prediction studies, classifying electrode channels as epileptogenic or not and categorizing operated patients as seizure-free or not.

These studies hold the potential to enhance the accuracy of identifying surgical targets by providing quantitative biomarkers that complement clinicians’ decision making. Improved accuracy in delineating epileptogenic networks can lead to more precise surgical planning and higher rates of post-surgical seizure freedom. Establishing reliable and reproducible prognostic biomarkers can reduce variability between clinicians and institutions, leading to more consistent outcomes across patient cohorts.

In this review, we provide an overview of how the studies published from 2017 to 2023 have been conducted. We evaluate the study designs and analytical approaches, discussing potential reasons for biomarkers not gaining traction. Our goal is to contribute to refining and standardizing qEEG study protocols, ultimately improving post-surgical outcomes for patients with drug-resistant epilepsy.

## 2. Methods

This review was registered on PROSPERO (ID: CRD42023382328), a database for pre-registering systematic reviews, to promote transparency and reduce bias. Thus, the review’s objectives and methodological approach were publicly documented before starting the data extraction, ensuring consistency and credibility throughout the review process. We adhered to the Preferred Reporting Items for Systematic Reviews and Meta-Analyses (PRISMA) guidelines [8].

### 2.1. Search Strategy

MEDLINE and Scopus were searched, using the PubMed and Elsevier search engines, for research and conference articles in English that were published from 2017 to 2023. Keywords were selected through an internal ballot among the research team, ensuring that the terms chosen would reflect the relevance to the study objectives. The search strategy was (epilepsy AND surgery) OR (epileptogenic AND network) OR (seizure AND onset AND zone) AND (eeg). The search syntax was adapted to each search engine (Table S1).

### 2.2. Eligibility Criteria

We included studies presenting an experimental method for invasive EEG analysis, aiming to complement clinicians in identifying the epilepsy surgery targets. Methods for predicting any of the theoretical concepts used as a proxy for epileptogenic brain area (seizure onset zone, surgical resection, alternative epileptogenic zone biomarker) were included. The validation had to be carried out on a cohort of two or more drug-resistant epilepsy patients (adult and pediatric) who underwent invasive EEG exploration. Exclusion criteria were the following: (i) using surface EEG, electrical source imaging or other imaging data, without invasive EEG; (ii) studies presenting post-surgical outcome prediction without predicting the surgical targets; and (iii) studies that did focus on surgical target prediction.

### 2.3. Outcomes

The main outcomes were surgery outcome classification (AUC, accuracy, sensitivity, specificity, F1-score, statistical tests (*p*-value), qualitative analysis), iEEG channel classification (same measures as above), comparison of outcomes stratified by the ground truth choice (seizure onset zone, resection, alternative epileptogenic zone biomarker), and distribution of the patient cohort parameters (cohort size, follow-up time, epilepsy types, outcome ratio, consecutiveness).

### 2.4. Data Extraction and Risk of Bias Assessment

Two reviewers (KI and AP) independently screened the abstracts to determine whether they met the eligibility criteria. The third reviewer (RZ) resolved any conflicting decisions. abstrackR web-application was used for screening the abstracts [9].

One reviewer (KI) performed the data extraction. The Systematic Review Data Repository-Plus (SRDR+) online platform was used for extracting, archiving, and sharing data between the authors. The project data can be found here [10], and data extraction is detailed in the Supplementary Materials. The main text of the selected studies was read to confirm the study’s eligibility. Main text and Supplementary Materials were used for data extraction. Extracted data included the approach summary, cohort size, cohort selection criteria, cohort source, good outcome reference (Engel or ILAE score), outcome ratio, epilepsy types, minimum post-surgical follow-up duration, ground truth reference, iEEG type, and performance scores. Missing data were recorded as missing.

Risk of bias (RoB) was assessed by the same reviewer using the QUAPAS (Quality Assessment of Prognostic Accuracy Studies) tool, which is specifically designed for prognostic studies [11]. The application of QUAPAS is detailed in the Supplementary Materials.

### 2.5. Data Synthesis

As studies are highly heterogeneous, qualitative analysis of the extracted data was performed, focusing on the approach summary, cohort characteristics, and method validation details. We employed a narrative synthesis to identify common patterns across studies, emphasizing the different methodological strategies and their reported outcomes. We categorized studies based on the type of EEG analysis methods employed, the ground truth reference utilized, and the quality of evidence presented. Statistical heterogeneity was explored through a careful examination of performance metrics, outcomes against the established ground truths, and patient cohort parameters. Subgroup analyses considered variations in study designs and cohort characteristics. Each subgroup was assessed for its impact on the performance metrics of surgical target identification and outcome prediction. Python 3.9.16 and the libraries Matplotlib 3.7.1, SciPy 1.10.1, NumPy 1.24.3, Pandas 1.5.3 and Matplotlib-venn 0.11.7 were used for visualizations and analyses. Miniconda3 23.7.2 was used for environment management.

## 3. Results

We designed a search strategy to encounter studies of interest to this review, using generally accepted keywords such as “epilepsy surgery”, “eeg”, “epileptogenic network”, and “seizure onset zone”. The search strategy identified 1798 publications (635 in Scopus, 1163 in MEDLINE), and 128 of these (7%) were included in the review (see PRISMA flowchart in Supplementary Figure S1). We avoided including keywords more specific to quantitative EEG analysis, such as “quantitative EEG”, since we noticed that no standard terms were used across studies.

### 3.1. Exploratory and Prediction Studies

The studies included in the analysis were retrospective prognostic studies investigating quantitative EEG markers that may identify the EN. Here, we use the EN term to refer to the surgical target and the theoretical concept of the epileptogenic brain network, as the reviewed studies used different references. Some studies additionally examined whether a marker could identify good and poor post-surgical outcomes. These studies could be categorized into exploratory (36%) and prediction studies (64%). Exploratory studies were further divided into those investigating whether a marker is associated with the EN (24%) or with post-surgical outcomes (12%). In contrast, prediction studies aimed to identify markers that can accurately predict the EN (46%) or post-surgical outcomes (18%).

**Figure 1:**
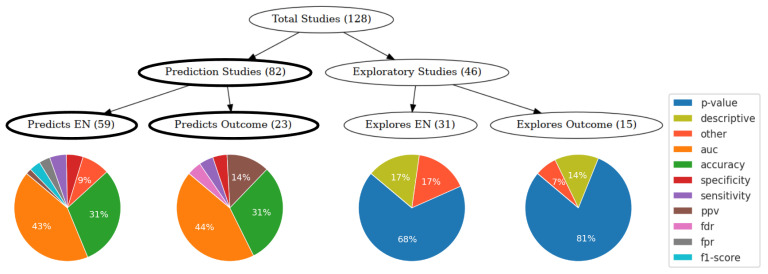
Flowchart for classifying the studies as exploratory or predictive. Studies are stratified according to their focus—epileptogenic network (EN) or surgical outcome. Proportions of study result metrics are shown for each group. The prediction studies (highlighted) are the main focus of the review. AUC—area under the receiver-operator-characteristic curve; ppv—positive predictive value; fdr—false discovery rate; fpr—false positive rate.

After stratifying the studies into exploratory and predictive categories, we observed significant heterogeneity in their analytical approaches and outcome metrics. Among exploratory studies, the main outcomes were typically expressed as statistical significance (*p*-value) or descriptive observations (Figure 1). Prediction performance in most studies was quantified using the area under the receiver-operating characteristic curve (AUC), followed by accuracy. Other metrics used were specificity, sensitivity, F1-score, positive predictive value, false discovery rate, and false positive rate (Figure 1).

### 3.2. Patient Cohort Properties

**Figure 2:**
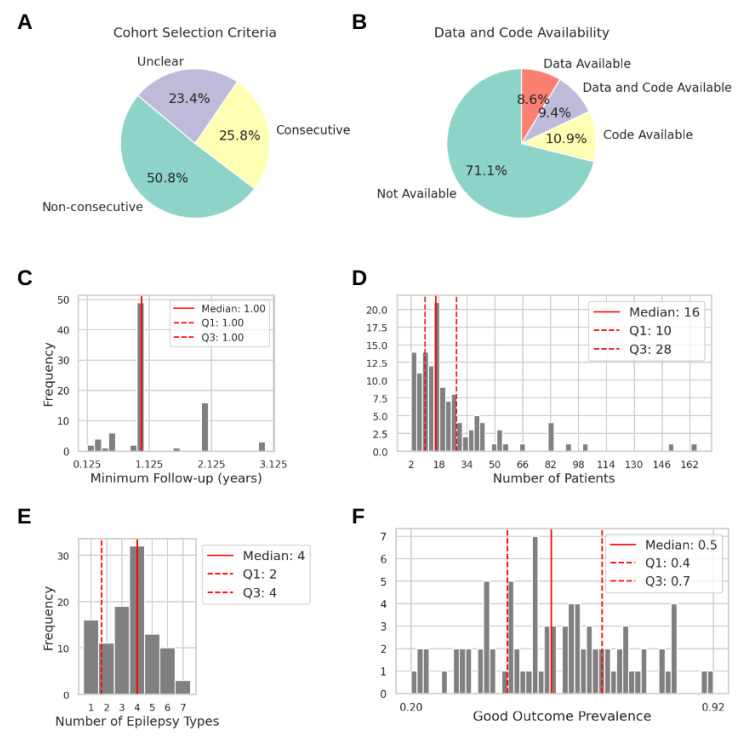
Cohort selection criteria and parameter distributions across the reviewed studies (n = 128). (**A**) Cohort selection criteria. (**B**) Data and code availability. (**C**) Histogram of the minimum post-surgical follow-up criterion (n = 84; 43 (33.6%) studies did not report minimum follow-up information or analyzed non-operated patients; Min = 0.125 years, Max = 3 years). (**D**) Histogram of the number of included patients (n = 128; Min = 2, Max = 166). (**E**) Histogram of the number of epilepsy types included (n = 105; 24 (18.8%) studies did not report epilepsy types; Min = 1, Max = 7). (**F**) Histogram of the good outcome prevalence across study cohorts (n = 105; 22 (17.2%) studies included non-operated patients undergoing iEEG monitoring; 20 (15.6%) studies only included patients with good outcomes; Min = 0.2, Max = 0.92).

Patient selection criteria and the final cohort properties varied widely across studies. We evaluated the cohorts as consecutive or non-consecutive to assess their representation of a realistic population of surgical candidates, similar to what would be expected in a clinical trial (see Supplementary Materials for evaluation details). A consecutive cohort with minimal selection criteria representing all surgical candidates from a center was reported in 33 studies (26%). A non-consecutive cohort with additional selection criteria excluding some surgical candidates was reported in 65 studies (51%). Thirty studies (23%) did not clearly report the selection criteria (Figure 2A). Most studies (71%) did not provide data or a code, while only 12 studies (9%) provided both data and a code (Figure 2B).

Most studies set the minimum post-surgical follow-up to 1 year (58%; 49 of 85 studies that reported follow-up information). Some studies (19%; 16/85) considered follow-up periods of less than 1 year, with the shortest being one and a half months, and the longest follow-up was 3 years (Figure 2B). In addition, 43 studies (34%) did not report follow-up information, and 22 studies (17%) included non-operated patients.

The number of patients ranged from 2 to 166 (Median = 16, Q1 = 10, Q3 = 28; Figure 2C). The number of epilepsy types varied from 1 to 7 (Median = 4, Q1 = 2, Q3 = 4; Figure 2D). Fifteen studies (12%) included only temporal lobe epilepsy. Twenty studies (16%) included only patients who were seizure-free after surgery (good outcome). The remaining studies had varying prevalence of good and poor outcomes, from 20% to 92% of good outcomes, with a median prevalence of 50% (balanced cohort; Figure 2E). Most cohorts thus differed from the real-world population, where the surgical success rate is 50–65%.

None of the studies estimated a sample size to assess the margin of error and general statistical robustness. Less than half of studies predicting post-surgical outcomes (7, 41%) used multi-center (external) validation.

### 3.3. Study Designs Are Heterogeneous Across Studies

**Figure 3:**
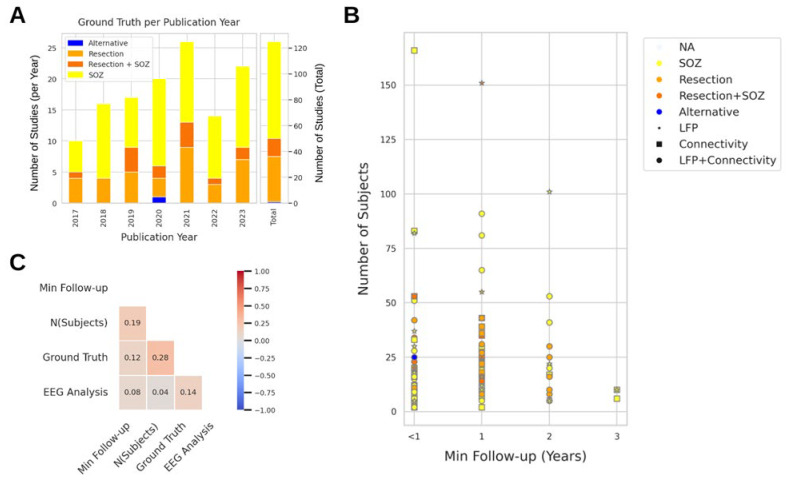
(**A**) Ground truth choice distribution across years. Alternative reference is shown in blue, resection in orange, resection and SOZ together in red orange, and SOZ in yellow. (**B**) Scatter plot showing the distribution of all studies relative to the number of subjects and minimum post-surgical follow-up time. The datapoint colors indicate the choice of ground truth (light blue—not available (NA), yellow—SOZ, orange—resection, red orange—resection and SOZ, blue—alternative), and the shape indicates the analytical approach (star—LFP, rectangle—connectivity, circle—LFP and connectivity). (**C**) Spearman’s correlation matrix of study design features. The minimum follow-up and the number of subjects are continuous features, while the ground truth and EEG analysis are discrete features. The minimum follow-up values were binned into four bins for clarity: x < 1, 1 ≤ x < 2, 2 ≤ x < 3, and x = 3 years. Discrete features were encoded into numerical values in the following fashion. For the ground truth: SOZ—1, resection—2, resection and SOZ—3, alternative—4. For the EEG analysis: LFP—1, connectivity—2, LFP and connectivity—3. Positive correlation between feature X and a discrete feature indicates that the larger X values appear more likely with a discrete variable encoded with a larger number.

Ground truth choices included the SOZ, the resection, both the SOZ and the resection, or an alternative reference. The SOZ was the most frequently chosen option throughout the years (Figure 3A), with 75 (59%) studies selecting the SOZ, 35 (27%) choosing the resection, and 14 (11%) opting for both the SOZ and the resection. One study, Guo and colleagues [12], validated their results against the epileptogenic zone as defined by the Epileptogenicity Index [13]. Three studies did not clearly state which reference was used.

The analytical approaches could be divided based on the iEEG analysis approach into LFP and connectivity studies (Figure S2). In total, 40 studies (31.3%) were LFP-based, 35 (27.3%) were connectivity-based, while 53 (41.4%) were a combination of the two. There were 24 (19%) studies based on high-frequency oscillations (HFOs). Fifty studies (39%) reported a computational model, and 40 (31%) used a machine learning approach to make predictions. Two studies analyzed electrical source imaging in addition to iEEG, while fifteen (12%) analyzed electrical stimulation data (Figure S2).

We illustrated the variation in the study designs by distributing studies based on the number of patients, minimum follow-up, ground truth selection, and the EEG analysis approach (LFP-based, connectivity-based, or a combination of both; Figure 3B). The study with the most patients (n = 166) had a minimum follow-up of 6 months and used a connectivity-based approach to identify the SOZ. The second-largest study (n = 151) had a 1-year follow-up and used an LFP-based approach to identify both the SOZ and the resection. Studies with 3-year follow-up periods had fewer patients, which can be explained by the limited number of patients at hospital centers and challenges with inter-center data sharing. There was no correlation found between any study design features (Figure 3C), further confirming the lack of standardization in the study design.

### 3.4. Prediction Studies: Study Designs Behind Prediction Scores

**Figure 4:**
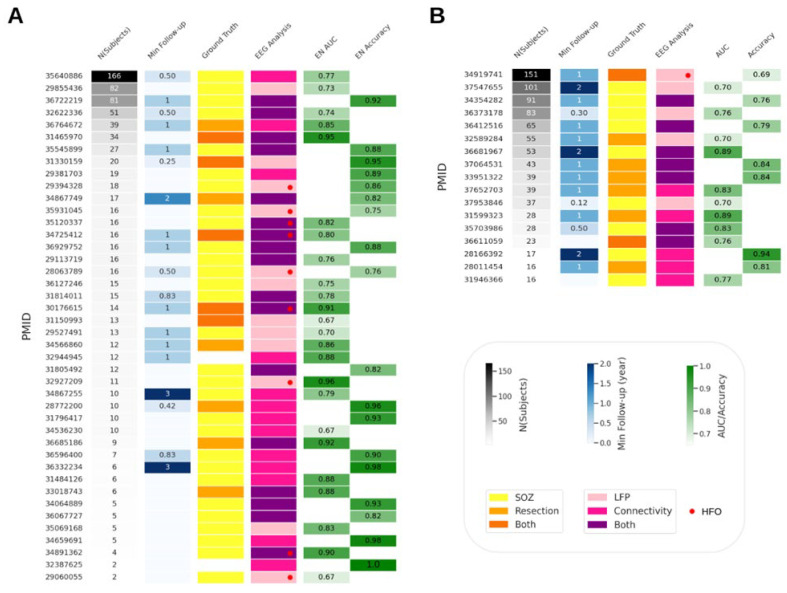
Prediction studies presenting the most common prediction metrics (AUC and accuracy)—study design. (**A**) Studies predicting the EN (EN prediction AUC mean = 0.81; STD = 0.09; min = 0.67; max = 0.96. EN prediction accuracy mean = 0.89; STD = 0.071; min = 0.75; max = 1). (**B**) Studies predicting surgical outcome (outcome prediction AUC mean = 0.78; STD = 0.074; min = 0.699; max = 0.89. Outcome prediction accuracy mean = 0.81; STD = 0.071; min = 0.695; max = 0.94). Studies’ PubMed identifiers (PMID) are presented as indices. From left to right, the columns present the number of subjects included in a study; ground truth (SOZ—yellow, resection—orange, SOZ and resection—red orange), EEG analysis approach (local field potentials (LFP)—pink, connectivity—lilac, or both—purple; high-frequency oscillations LFPs are depicted as a red dot), AUC, and accuracy.

Ideally, different markers could be ranked by their predictive performance. However, due to the high heterogeneity in the study design (Figure 2) and outcome metrics (Figure 3), direct comparisons are challenging. Exploratory studies are crucial for identifying potential biomarkers, but they do not assess prognostic potential. For this reason, we focus on prediction studies in the main text, while details on exploratory studies are available in the Supplementary Figure S2. To facilitate comparisons, we concentrate on studies with the most reported metrics (AUC and accuracy; Figure 1). Comparing markers based solely on performance metrics can be misleading. Although most studies aim to generalize their approach for any type of drug-resistant epilepsy, marker performance is often tested on specific cohorts, leading to confounding effects such as underrepresentation of epilepsy types, small cohort sizes, and short post-surgical follow-up periods. Figure 4 presents the study design features for prediction studies reporting AUC or accuracy, focusing on either the EN (42 studies) or post-surgical outcome prediction (17 studies).

**Figure 5:**
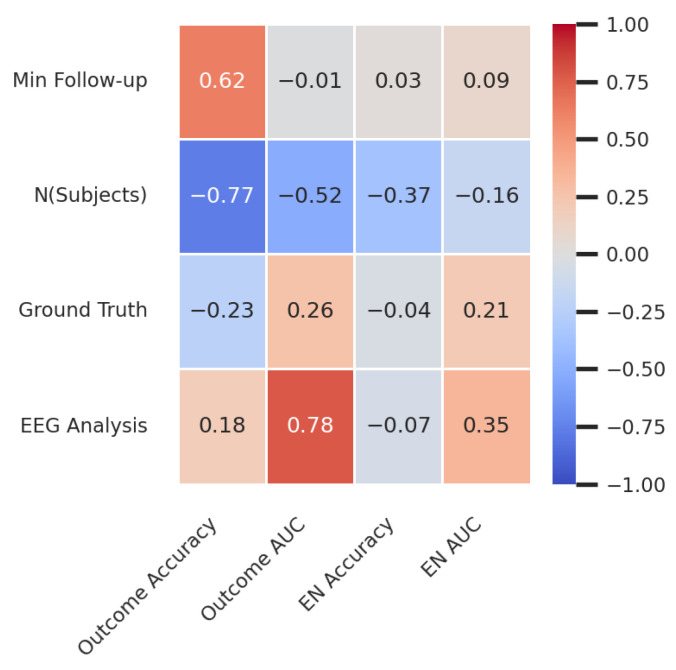
Spearman correlation matrix between the prediction performance and study design features. The minimum follow-up and the number of subjects are continuous features, while the ground truth and EEG analysis are discrete features. As in Figure 3, discrete features were encoded into numerical values in the following fashion. For the ground truth: SOZ—1, resection—2, resection and SOZ—3; for the EEG analysis: LFP—1, connectivity—2, LFP and connectivity—3. Positive correlation between feature X and a discrete feature indicates that the larger X values appear more likely with a discrete variable encoded with a larger number.

For the EN studies (Figure 4A), the number of subjects ranged from 2 to 166 (median = 13). Follow-up periods varied from 3 months to 3 years, with 7% of studies not providing follow-up information and 46% using non-operated cohorts. In terms of analytical approach, 32% were connectivity-based, 29% LFP-based, and 49% a combination of both (including six HFO studies). The highest AUC score (0.96) was achieved using an HFO-based analysis in a cohort of 11 patients without follow-up information, using the SOZ as ground truth. The study with the largest cohort (166 patients with a minimum 6-month follow-up) proposed a connectivity-based marker for predicting the SOZ, achieving an AUC of 0.77. Perfect accuracy was reported in a cohort of two patients without clear follow-up or ground truth information. Two studies used cohorts with a 3-year minimum follow-up, yielding the most stable long-term results, as the probability of seizure recurrence decreases over time. These studies reported an accuracy of 0.94 and an AUC of 0.79 in cohorts of 6 and 10 patients, respectively, using the SOZ as ground truth and a connectivity-based approach. There was a slight negative correlation between the number of subjects and the accuracy of EN prediction (Spearman correlation coefficient for AUC = −0.16; for accuracy = −0.37; Figure 5). Connectivity-based studies were slightly more likely to obtain higher AUC scores than the LFP-based approaches (Spearman’s coefficient = 0.35; Figure 5).

For post-surgical outcome prediction, the accuracy of a marker tested on the largest cohort (166 patients) was 0.69, which is close to the current state of the art (visual analysis). The highest accuracy (0.94) was achieved in a study with 17 patients. Higher outcome prediction scores were more likely in smaller cohorts (Spearman’s coefficient for AUC = −0.52; for accuracy = −0.77; Figure 5). Longer follow-up periods were positively correlated with higher outcome prediction accuracy (Spearman’s coefficient = 0.62). Connectivity-based approaches were also more likely to provide better outcome predictions than the LFP-based approaches (Spearman’s coefficient = 0.78).

### 3.5. Risk of Bias Assessment

Risk of bias was assessed using the QUAPAS tool [11] in five domains—participants, index test, outcome, flow and timing, and analysis. We note the criteria for answering the QUAPAS questions in the Supplementary Materials to enhance the reproducibility of the assessment, since the assessment was conducted by a single reviewer. The selection of patients was rated as high-risk in 52% of studies, while unclear in 9% (Figure S5—Domain 1). The index test was rated as high-risk if the outcome was known before (such as in exploratory studies), which was the case in 27% and unclear in 4% of studies (Figure S5—Domain 2). Outcome measurement was rated as high-risk in 5% of studies and unclear in 2% (Figure S5—Domain 3). Flow and timing were rated as high-risk in 33% because of a short post-surgical follow-up (less than 1-year) and unclear in 15% because of unavailable follow-up information (Figure S5—Domain 4). The analysis was rated as high-risk if not all patients were enrolled in the analysis, which was the case in 53% of studies (Figure S5—Domain 5).

## 4. Discussion

This review investigated exploratory and prediction studies in qEEG research, with a specific focus on identifying effective markers of ENs in drug-resistant epilepsy. Despite more than 30 years of research, to the best of our knowledge no qEEG markers have been implemented in clinical practice [14]. Our findings indicate that connectivity-based markers show more promise to predict outcomes than the LFP-based approaches. However, high variability in study designs and analytical approaches used to validate the study result represent an important weakness in the field. This diversity makes direct comparisons between the prognostic value of biomarkers difficult. Differences in outcome metrics used across studies further hinder straightforward ranking of biomarkers. Even when outcome metrics align, variation in validation protocols—such as follow-up times and ground truth definitions—complicates comparisons, as the same metric may reflect different validation criteria. Consequently, our meta-analysis was limited to small subgroups of studies with shared features. These methodological inconsistencies likely contribute to the slow adoption of most biomarkers for clinical application. We discuss the challenges and propose potential strategies to facilitate the translation of promising research findings into clinical applications.

### 4.1. Study Design Standardization

The qEEG studies are highly heterogeneous, with variations in study design and analytical approach. This variability complicates direct comparisons and reproducibility and increases the risk of bias (Figure S4). Furthermore, data sharing is limited, as 82% of studies do not share data due to anonymity concerns (Figure 2B), and only 20% of studies make their codes available, restricting further development beyond initial publication. Only a few methods have gained traction and follow-up publications, such as EI [15], Virtual Epileptic Patient (VEP) [16], and high-frequency oscillations (HFOs) [17]. Only VEP is currently undergoing a clinical trial (EPINOV; clinical trial identifier: NCT03643016 [18]). Despite common strategies to design a study, individual studies differ greatly due to patient cohort characteristics and non-standardized validation strategies. Although many studies report high prediction accuracies, none of these methods have been adopted into clinical practice.

Designing a robust qEEG study requires navigating both uncontrollable and controllable factors. Uncontrollable factors include the theoretical nature of qEEG concepts, which are difficult to prove and substantiate empirically, as well as the limited number of available patients and obstacles associated with data sharing. Additionally, inherent variability in cohort characteristics such as the types of epilepsy and the range of patient outcomes preclude standardization. On the other hand, controllable factors include the choice of analysis approaches, such as performance scores for both epileptogenic zone (EZ) and outcome prediction, as well as the selection of ground truth references and patient selection criteria. Rigorously addressing these controllable factors can enhance the robustness and applicability of study results.

#### 4.1.1. Patient Cohort Characteristics

The patient cohort characteristics are fundamental for interpreting the results of any study. A robust study requires a large cohort to represent the population of drug-resistant epilepsy patients. Retrospective studies offer advantages such as cost-effectiveness, long-term outcome insights, and the ability to collect large datasets, enhancing statistical power and robustness [19]. However, selection criteria must be sufficient to ensure generalizability across all drug-resistant epilepsy patients and to reflect the real-world patient population. We found that the best outcome prediction performance was achieved in smaller cohorts, with a negative correlation between cohort size and outcome prediction performance. The reviewed study designs always lacked previous sample size calculation. Moreover, some cohorts had an imbalanced prevalence of good and poor surgical outcomes, rather than a representative sample. As a result, the findings might not accurately reflect the true variability and effectiveness of a marker, leading to greater uncertainty in the study’s conclusions and making it harder to generalize the results to a larger population [20]. A large cohort that is representative of a real-world clinical situation can be achieved by combining cohorts from multiple centers. Cohorts should ideally include a variety of epilepsy types and sufficient follow-up duration to provide meaningful results. There are works in which the authors focus on one epilepsy type, mostly temporal epilepsy, or on a specific pathology, such as focal cortical dysplasia, in which case the selection criteria are adjusted. However, the works should clearly state at which population a marker is aimed. Additional subgroup analyses can be provided to test the marker in a case–control manner, relative to the epilepsy type, age, or other clinical parameters.

#### 4.1.2. Ground Truth Choice

A promising marker would distinguish between the EN nodes (iEEG electrodes) and the non-EN nodes. On the level of data collection, the field faces an unavoidable spatial bias due to the implantation of a limited number of electrodes. As we cannot sample the entire brain, we must rely on a restricted representation of the patient’s brain network, which may or may not capture the relevant nodes. Also, the EN concept is theoretical and challenging to validate. Ground truths such as alternative qEEG markers, the seizure onset zone (SOZ), and the surgical resection area in patients with good surgical outcomes are commonly used. However, these references are not homologous, and the choice of ground truth significantly influences the study’s results. It is essential to clearly disclaim the chosen ground truth within a study’s objectives to ensure clarity and understand its relevance. Studies based on alternative references are useful for exploration, but only indirectly related to the SOZ and the resection. Most of the studies we revised opted for the SOZ (Figure 3A), as determined by visual analysis of the iEEG. As such, the SOZ is liable to variability of criteria between clinical specialists. This subject was discussed in a recent review by Flanary and colleagues [21], where the authors caution about the uncertainty in defining the SOZ among clinicians and highlight the inherent limitations in the accuracy of any algorithmic approach that uses the SOZ as a ground truth for performance comparison. Studies based on the SOZ may provide an algorithm for automation of SOZ identification, which could eliminate some of the variability. However, the SOZ is rarely fully resected (39.3–97.6% of SOZ resected), and the proportion of resected SOZ does not correlate with seizure freedom, not even when the entire SOZ is removed [22]. While the SOZ is crucial for surgical planning, the resected areas are more directly related to patient outcomes. The long-term post-surgical seizure freedom proves that a resection indeed included the EN. Thus, studies based on the surgical resection aim to predict the epileptogenic network, advise the surgical target, and consequently contribute to improving surgical outcomes. Nevertheless, the surgery must preserve essential brain functions. The final surgical resection may thus include non-epileptogenic nodes, which would be considered as false positives by an accurate algorithm. Accurate validation requires considering both SOZ and resected areas, as discrepancies between them can lead to identifying incomplete or non-feasible surgical targets.

#### 4.1.3. Post-Surgical Outcome Prediction: Follow-Up Time and Analysis Approach

Most prediction studies focus on identifying the EN (Figure 1), without investigating the differences between good and poor post-surgical outcomes. Some studies focus only on seizure-free patients because surgical target predictions in non-seizure-free patients cannot be validated. While simplifying the approach for exploratory purposes is reasonable, the prognostic accuracy of a marker reflects its ability to distinguish between post-surgical outcomes. It is crucial to consider the timing of outcome registration. A 1-year follow-up is commonly used. However, 15–70% of patients relapse after the first year, depending on resection location, with recurrence probability decreasing each following year [2]. Longer follow-ups provide more stable outcome information and, consequently, more reliable results. However, those are available for fewer patients, potentially leading to data loss. If the follow-up restrictions result in a small cohort, the rarer epilepsy types may not be represented. Extra-temporal epilepsies comprise approximately 25% of patients [23], with particularly infrequent types like insular epilepsies. Thus, smaller cohorts are more likely to overrepresent temporal epilepsy, potentially skewing the conclusions. In this case, the generalizability of the approach should be discussed. To maximize available data, we suggest analyses at multiple follow-up timepoints. This would also provide additional insights into marker applicability, e.g., some markers may be predictive of short-term outcomes, while other markers may predict long-term seizure freedom.

We observed that there is no general agreement on the biomarkers so far. Many studies advocate for HFOs; however, this review reveals that they do not exceed other approaches. Network-level biomarkers have been becoming a focus in recent years. Despite the variability in approaches, it is important to note that the results across studies do not contradict, but likely capture different aspects of the EN and may, therefore, complement each other in the common goal of describing the complex EN dynamics. Both LFP- and connectivity-based markers achieved high prediction performances. We found, however, that connectivity-based markers were advantageous in predicting both ENs and surgical outcomes (Figure 5). Still, we were unable to compare the markers due to the high heterogeneity of analytical approaches and study outcome metrics. Most studies do not assess the capability of the marker to predict post-surgical outcomes. Even after stratifying the studies based on whether they performed post-surgical outcome prediction tests, substantial variability in analytical approaches and performance metrics persisted. Some studies (31%, Figure S2) used machine learning techniques to evaluate the predictive capacity of markers. In these machine learning-dependent approaches, the prediction results were closely tied to the training set, introducing variability. Conversely, other studies assigned a quantitative value to each patient, which non-learning classifiers used to statistically separate the classes. These studies quantified whether the qEEG marker value of the EN nodes (a node score) differed from the non-EN nodes, and whether the EN correspondence to the ground truth differed between good and poor post-surgical outcomes. The area under the receiver operating characteristic curve (AUC) is the most frequently reported prediction metric, appearing in 46% of prediction studies (Figure 1). AUC is based on the rank order of the node scores rather than their actual values, so it is independent of the scale or distribution of the scores. This makes it an effective metric for evaluating the prediction performance in various contexts, including qEEG studies. We further noted that only one study [24] validated the marker against a null model. This approach ensures that markers do not identify the same nodes in a random network, confirming that detected differences are not merely due to random data variations unrelated to seizure generation. Ideally, an algorithm should return a negative result if none of the nodes are epileptogenic. Additionally, developing open-source tools and promoting data sharing are crucial for enhancing transparency, reproducibility, and collaborative development in the field, as recently discussed by Bernabei and colleagues [25]. Müller and colleagues [26] are among the few that have reviewed and compared two qEEG methods, highlighting the need for more comprehensive evaluations and standardization in this research area.

**Figure 6:**
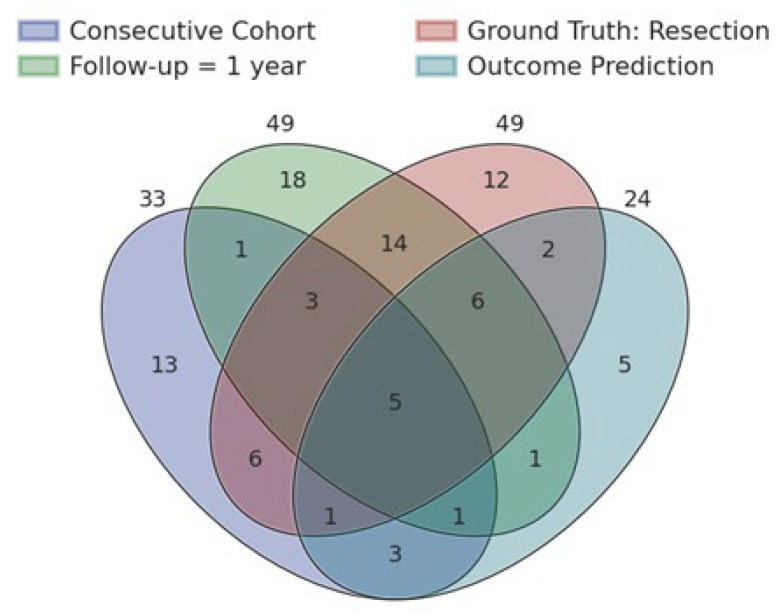
Venn diagram of study design features, ensuring minimum bias. These features are a consecutive cohort (purple) with a minimum follow-up of 1 year (green), considering the resection as ground truth (red), and performing surgical outcome prediction (blue).

Following the discussion above, we consider the key features that make a robust study which would be replicative and as close to the clinical trial as possible. These include (i) a consecutive cohort with a long follow-up, (ii) minimally 1 year, but preferably longer), (iii) validation with the resection (not only the SOZ), and (iv) post-surgical outcome prediction. Out of 128 studies, only 5 included these features (Figure 6). These studies provide examples of a robust study design. For instance, Matarrese and colleagues [27] studied the clinical value of the spike onset as a biomarker. The validation cohort was consecutive, with minimal selection criteria: the availability of long-term monitoring with intracranial EEG; pre- and post-surgical MRI and post-implant CT; SOZ defined by clinicians in the presurgical phase; and availability of surgical outcome at least 1 year after surgery. The cohort consisted of 43 patients, including frontal, temporal, parietal, occipital, insular, and multilobar epilepsies. The authors compared the brain areas defined by the biomarker to the surgical resection and tested the prediction of post-surgical outcome based on the resection of these areas. The average resection overlap with the spike onset zone in good outcome patients (Engel I) was 96%, while in poor outcomes, the resection was almost completely missed. The resection of the spike onset zone yielded surgical outcome prediction with a PPV of 79%, an NPV of 56%, and an accuracy of 69%. The authors concluded that the surgical resection of the spike onset disrupts the EN and may ensure seizure freedom. Including these four features in their study design provides robustness and generalizability of the study’s insights across drug-resistant epilepsy patients at least 1 year post-surgery. Increasing the follow-up limit to 2 years, none of the studies fit the criteria (Figure S4).

## 5. Conclusions

The development of qEEG tools for guiding epilepsy surgery is inherently hindered by the variability in drug-resistant epilepsy patients and lack of understanding of the complex dynamics of brain networks. Studies face the challenge of not having a ground truth and must work with rigorous statistical analyses to validate their tool. This review revealed potential gaps in study designs that may impede the advancement of the field.

## 6. Future Directions

Without underestimating the difficulty of the task, we propose the standardization of the study design, which would minimize confounding effects and maximize the result validity. Standardizing study designs, along with addressing data- and code-sharing challenges, would facilitate the testing, replication, and external validation of promising markers. These implementations would move the field closer to integrating qEEG markers into clinical practice, ultimately improving the rate of seizure freedom in drug-resistant epilepsy patients.

## Data Availability

The extracted data and the Jupyter notebook to reproduce results is available on GitHub (https://github.com/ivkarla/epirev, accessed on 17 October 2024).

## Supplementary Materials

The following supporting information can be downloaded at: https://www.mdpi.com/article/10.3390/biomedicines12112597/s1, Supplementary Methods contain a detailed protocol of data extraction using the SRDR+ extraction form. Supplementary Results: Figure S1: PRISMA flowchart of study selection; Figure S2: Summary of analytical approaches; Figure S3: Exploratory studies presenting statistical comparison between groups—study design and *p*-values; Figure S4: Venn diagram of study design features ensuring minimum bias; Figure S5: RoB assessment using the QUAPAS tool.
