# Supplementary material for: Methods for Identifying Epilepsy Surgery Targets Using Invasive EEG: A Systematic Review
Source: Biomedicines. 2024 Nov 13;12(11):2597. doi: 10.3390/biomedicines12112597 (PMC11592023; doi:10.3390/biomedicines12112597)
Supplement: Supplementary file 1 [file biomedicines-12-02597-s001.zip › biomedicines-3293410-supplementary.pdf]

biomedicines

MDPI

Supplementary Methods

Methods for Identifying Epilepsy Surgery Targets from Invasive EEG: A Systematic Review

Karla Ivankovic [1,2], Alessandro Principe [1,2], Riccardo Zucca [1,2], Mara Dierssen [1,2,3,4] and Rodrigo Rocamora [1,2]

[1] Universitat Pompeu Fabra (UPF), Barcelona, Spain
[2] Hospital del Mar Research Institute, Barcelona, Spain
[3] Centre for Genomic Regulation (CRG), Barcelona, Spain
[4] Biomedical Research Networking Center on Rare Diseases (CIBERER), Institute of Health Carlos III, Madrid, Spain

Search strategy

Table S1. Search strategy for databases searched.

Database	Search string	Date of search	# of results
Scopus	TITLE-ABS-KEY((epilepsy AND surgery) OR (epileptogenic AND network) OR (seizure AND onset AND zone) AND (eeg) AND (LIMIT-TO(SRCTYPE, “j”) OR (LIMIT-TO(SRCTYPE, “b”) OR (LIMIT-TO(SRCTYPE, “k”) or (LIMIT-TO(DOCTYPE, “ar”) OR (LIMIT-TO(DOCTYPE, “cp”) AND (LIMIT-TO(PUBYEAR, 2017-2023)) AND (LIMIT-TO(LANGUAGE, “English))	08/01/2024	635
MEDLINE	(((epilepsy) AND (surgery)) OR ((epileptogenic)) AND (network)) OR (seizure AND onset AND zone) AND (eeg))) Filters: Classical Article, Clinical Conference, Clinical Study, Clinical Trial, Comparative Study, Congress, Corrected and Republished Article, Evaluation Study, Multicenter Study, Observational Study, Pragmatic Clinical Trial, Validation Study, Humans, English, Exclude preprints, from 2017 - 2023	08/01/2024	1163

Data extraction

The Systematic Review Data Repository-Plus (SRDR+) online platform was used for extracting the data. The questions addressed by the review were:

1. What are the existing methods for identifying epilepsy surgery targets?
2. What are the results and how do they compare to the current gold standard (trained experts)?
3. What is the quality of method validation, considering the patient cohort, ground truth reference, and validation metrics?
4. Does the study provide source code and data to replicate the results?

Design details

The following data was extracted:

1. Analytical approach description
2. Performance score (metric and value)

3. Data and code availability (Multiple choice: Data available, Code available, Not available (NA))
4. Cohort size (Total, Good outcome patients, Poor outcome patients)
5. Cohort selection criteria (Multiple choice: Consecutive, Non-consecutive, Non-operated, Unclear)
6. Good outcome reference (Multiple choice: Engel I, Engel I/II, ILAE 1, ILAE 1/2, Not operated, NA)
7. Epilepsy types (Multiple choice: Temporal, Frontal, Occipital, Parietal, Multilobe, Hemispheric, Insular/opercular, Premotor, Neocortical, Central, NA)
8. Follow-up (Information available or NA, Minimum follow-up, Follow-up for each patient)
9. Ground truth (Multiple choice: SOZ, Resection, Alternative reference, NA)
10. iEEG type (Multiple choice: SEEG, ECoG)

The data was considered unavailable if it could not be openly accessed without contacting the authors.

Patient selection criteria varied across studies. Minimal selection criteria to include all surgical candidates were consecutive patients from a single or multiple centers, who underwent pre-surgical iEEG monitoring, and had a defined SOZ. Additional criterion for studies that analyzed seizure activity was the availability of a recording of at least one seizure. Additional inclusion criteria for studies that considered the resection area as the ground truth was the availability of the anatomical localization of electrodes using post-implant MRI co-registered to the patient's pre-implant MRI, and additional exclusion criteria was hemispherectomy and non-resective surgery. If any additional selection criteria were listed, the cohort was considered non-consecutive (such as restrictions on epilepsy types, minimal sampling frequency, number of recorded seizures, psychometrics, seizure type, focality, Engel score and availability or a lack of specific electrophysiological activity, e.g., interictal discharges or gamma activity at seizure onset). If the selection criteria were not clearly listed, we noted it as unclear.

Outcomes

Type of study outcome (categorical or continuous), domain (statistical test or prediction performance), specific measurement and units were extracted. In case of multiple measurements, all outcomes were extracted.

Risk of bias (RoB) assessment: QUAPAS tool

QUAPAS tool consists of five domains evaluating the participants, the index test, the outcome, the timing and flow, and the analysis. Domains consist of a set of Yes/No/Unclear "signaling" questions to help judge RoB, and Low/High/Unclear "rating" questions. Some questions were ambiguous, and some were not applicable to the review. We define the criteria for answering the ambiguous questions and reasons for exclusion of non-applicable questions. The questions not listed here were non-ambiguous.

Domain 1: Participants

The RoB for the selection of participants was rated as High if the epilepsy types did not reflect the realistic patient population. The reference prevalence of different epilepsy types per lobe were 60% for temporal, 20% to 40% for frontal, 6% for parietal, and 5% to 10% for occipital lobe. If a cohort notably deviated from the reference, we considered a bias risk. This excluded studies that clearly stated that the study focuses on specific epilepsy types, and not drug-resistant epilepsy in general. Additionally, we considered a bias

risk in studies that included exclusively seizure-free patients, and studies that included patients with specific iEEG properties.

Domain 2: Index Test

The question (2.1) "Was the method used to perform the index test valid and reliable?" was excluded since most studies are the first report/proposal of the index test. The question (2.3) "Were the index test results interpreted without knowledge of the outcome?" was answered Yes if the study used the index test values to make a prediction of EN or post-surgical outcome. We considered observer bias risk of the index test interpretation and conduct if the outcome was known before (such as in exploratory studies).

Domain 3: Outcome

We considered the RoB of the measurement of the outcome if the outcome was measured at less than 1-year post-surgery. If the follow-up information was not available, it was rated as Unclear.

Domain 4: Flow and Timing

The question (4.3) "Was the time horizon sufficient to capture the outcome?" was answered Yes if the post-surgical follow-up was at least 1 year. The study flow was considered to have introduced bias if any of the signaling questions was answered with Yes. The concerns that the time horizon does not match the review question was High if non-operated patients were included.

Domain 5: Analysis

The questions (5.3) "Were appropriate methods used to account for censoring?" and (5.4) "In case of competing events, were appropriate methods used to account for them?" were excluded as they did not apply to the reviewed studies. RoB in the analysis was rated as High if not all patients were enrolled in the analysis.

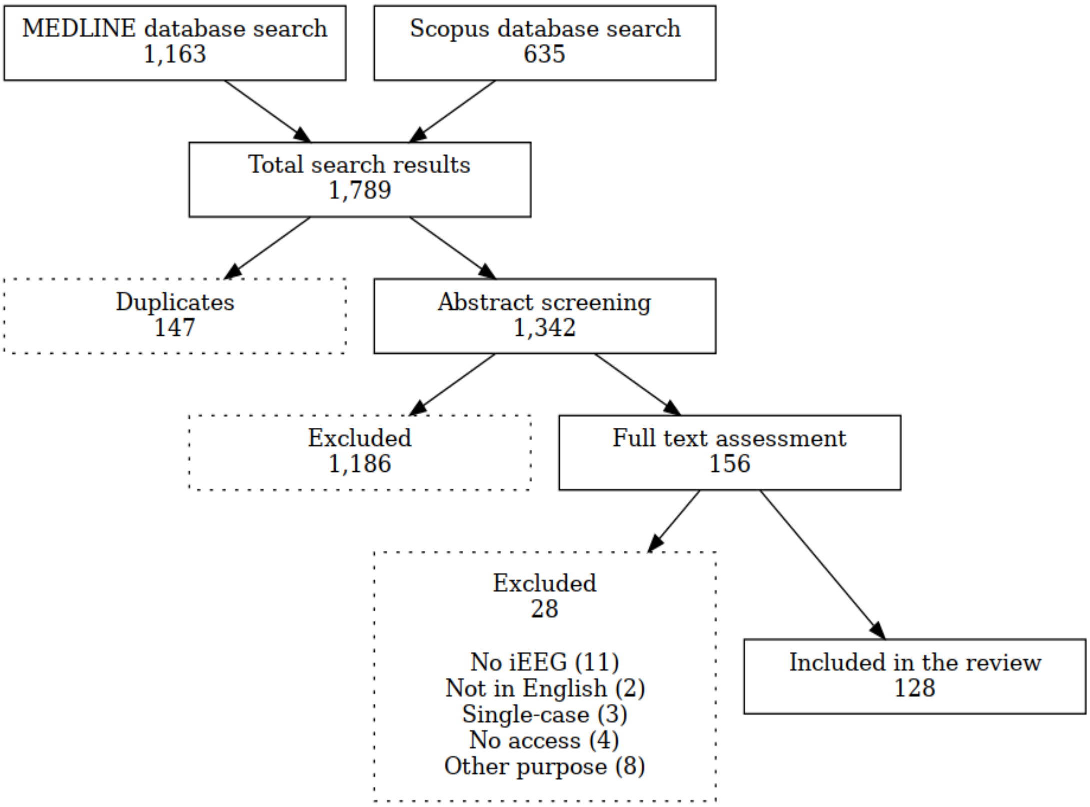

Figure S1. PRISMA flowchart of study selection.

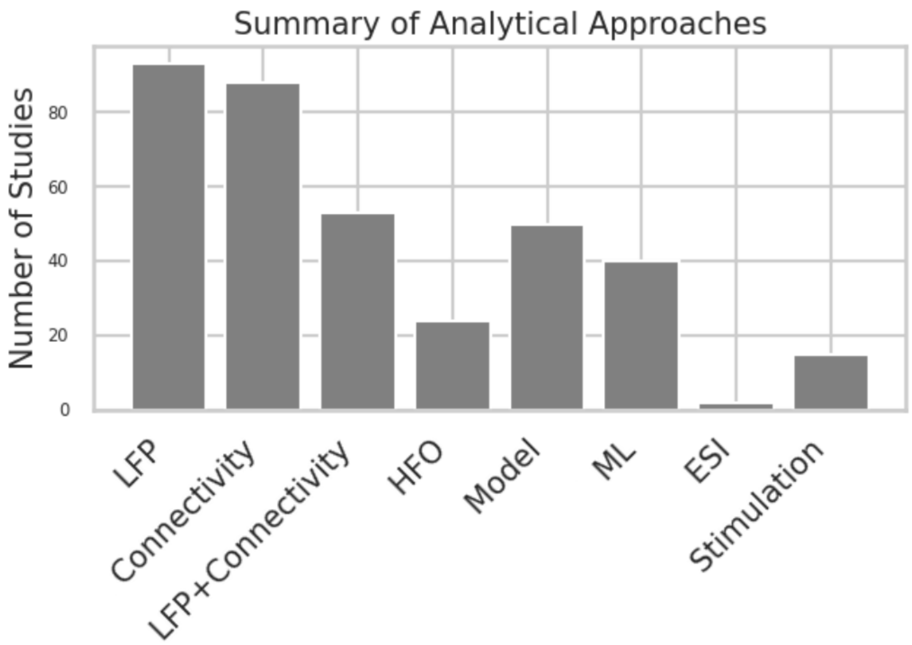

Figure S2. Summary of analytical approaches. The categories are overlapping (e.g., a study proposing a computational network model would be in categories "Connectivity" and "Model"). Out of 128 studies, 93 (73%) reported an LFP-based qEEG marker, 88 (69%) reported a connectivity-based marker. 24 (19%) were HFO-based, and 53 (41%) were combinations of both LFP and connectivity markers. Fifty studies (39%) reported a computational model, and 40 (31%) were using a machine learning (ML) approach to make predictions. Two studies were comparing iEEG data to electrical source imaging (ESI), while 15 (12%) were analyzing electrical stimulation data.

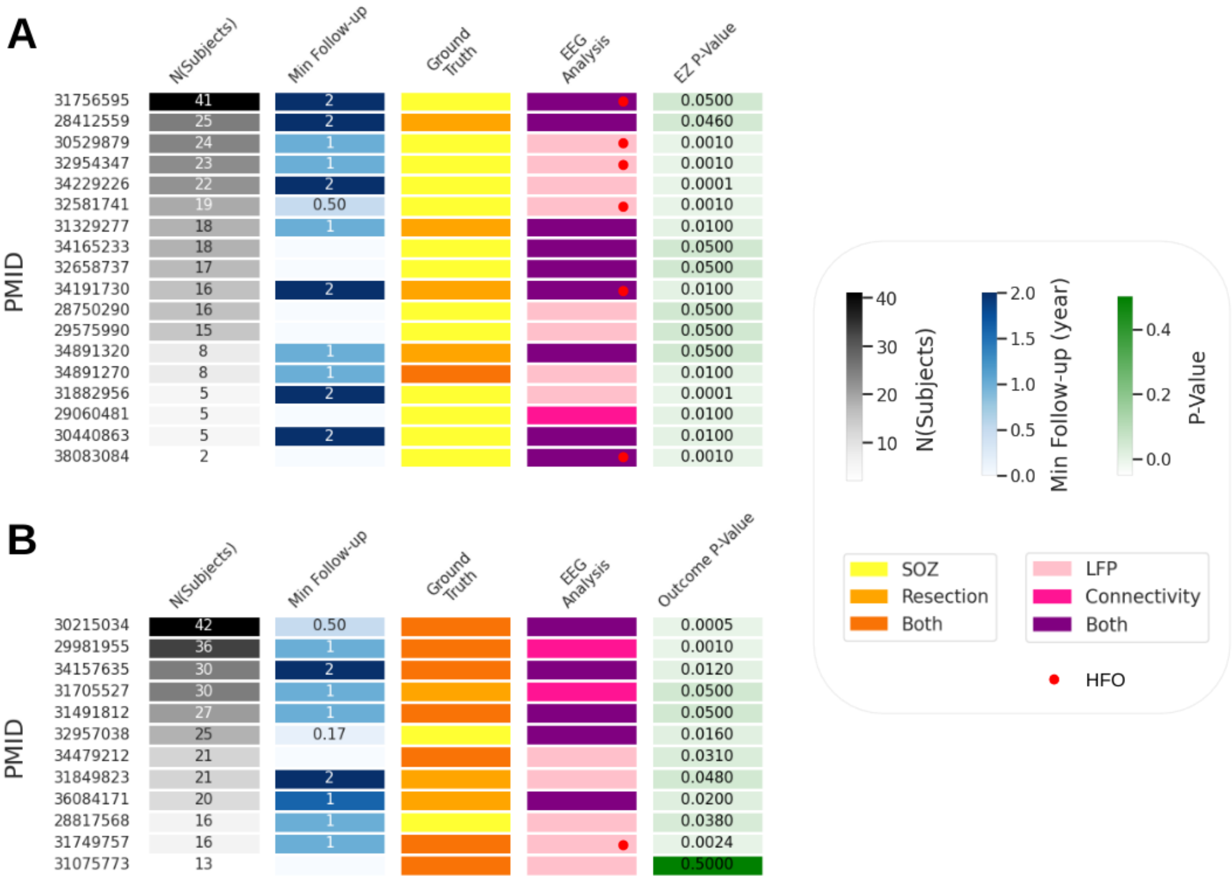

Figure S3. Exploratory studies presenting statistical comparison between groups - study design and P-values. (**A**) Studies focusing on comparing the epileptogenic zone (EZ) and non-EZ. (**B**) Studies focusing on comparing good and poor surgical outcomes. Studies' PubMed identifiers (PMID) are presented as indices. From left to right, the columns present the number of subjects included in a study; ground truth (SOZ - yellow, resection - orange, SOZ and resection - red orange), EEG analysis approach (local field potentials (LFP) - pink, connectivity - lila or both - purple; high frequency oscillations LFPs are depicted as a red dot), and P-value. Figure S3 shows study design features for the group of exploratory studies that presented the main result as a P-value, either focusing on the EZ (18 studies) or post-surgical outcomes (12 studies). All but one achieved a significant result, indicating association of a marker with an outcome. For the EZ studies (Fig S3A), the number of patients varied from 2 to 41 (Median = 17). The minimum follow-up time varied from 6 months to 2 years, while 33% studies used non-operated patients, so the surgical outcome was unavailable. Most used ground truth was the SOZ (72%). Most analyzed the LFPs (44%) of the combination of LFPs and connectivity (50%), a third of which were high-frequency oscillation (HFO) studies. For the post-surgical outcome studies (Fig S3B), the number of patients varied from 13 to 42 (Median = 23). The follow-up varied from 2 months to 2 years, with two studies not providing this information. In this case, the resection was the most represented ground truth (25% resection, 58% resection and SOZ; 17% SOZ). Again, the LFP-focused analysis was most common (83%; 1 HFO study).

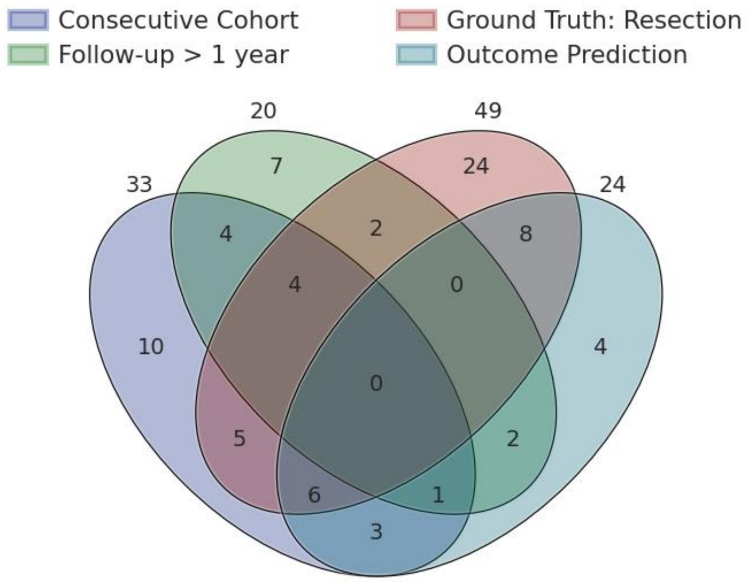

Figure S4. Venn diagram of study design features ensuring minimum bias. These features are a consecutive cohort (purple) with a minimum follow-up higher than 1 year (green), considering the resection as ground truth (red), and performing surgical outcome prediction (blue).

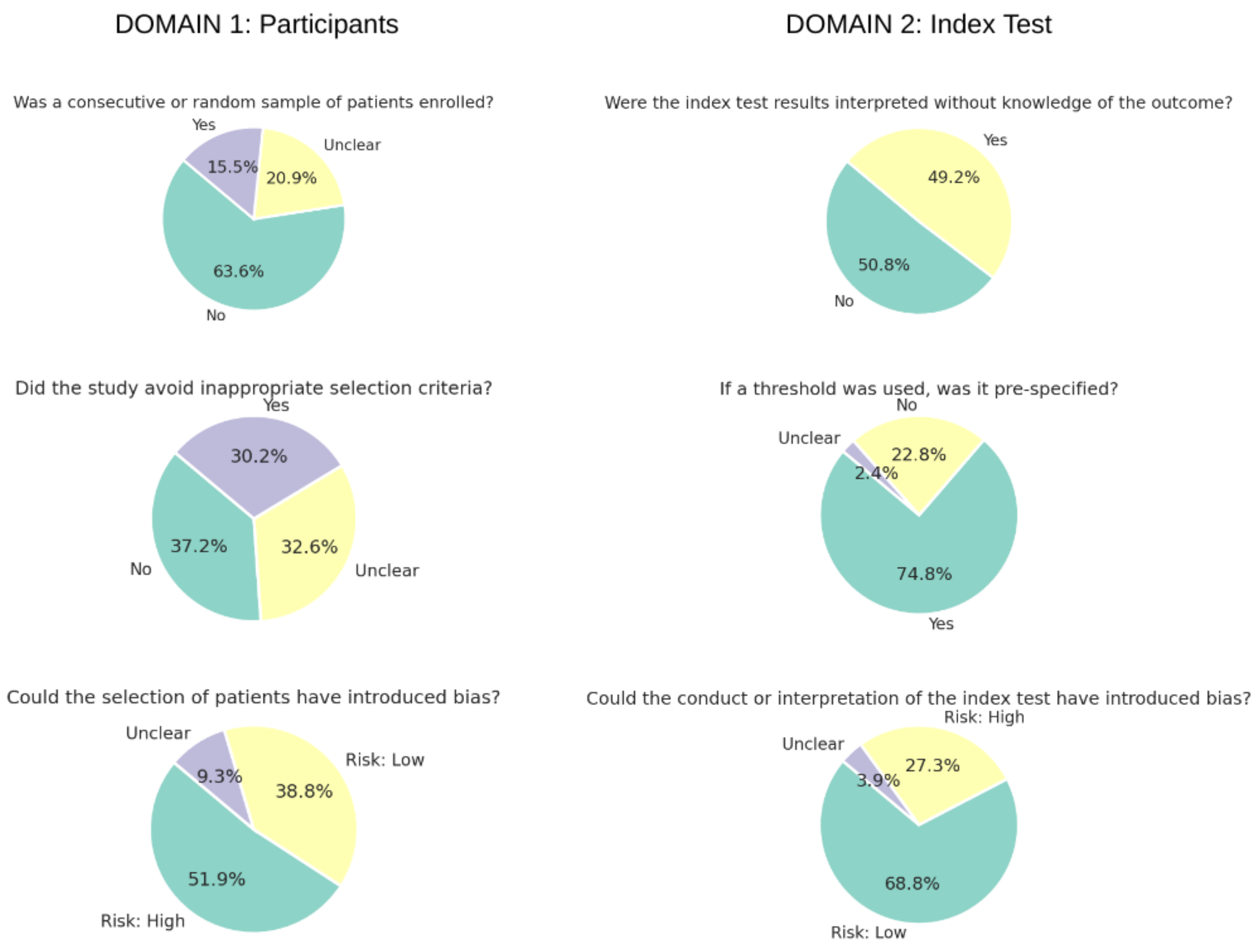
DOMAIN 1: Participants
DOMAIN 2: Index Test
Was a consecutive or random sample of patients enrolled?
Yes
Unclear
15.5%
20.9%
63.6%
No
Were the index test results interpreted without knowledge of the outcome?
Yes
49.2%
50.8%
No
Did the study avoid inappropriate selection criteria?
Yes
30.2%
37.2%
32.6%
No
Unclear
If a threshold was used, was it pre-specified?
No
Unclear
22.8%
2.4%
74.8%
Yes
Could the selection of patients have introduced bias?
Unclear
9.3%
38.8%
Risk: Low
51.9%
Risk: High
Could the conduct or interpretation of the index test have introduced bias?
Risk: High
Unclear
27.3%
3.9%
68.8%
Risk: Low

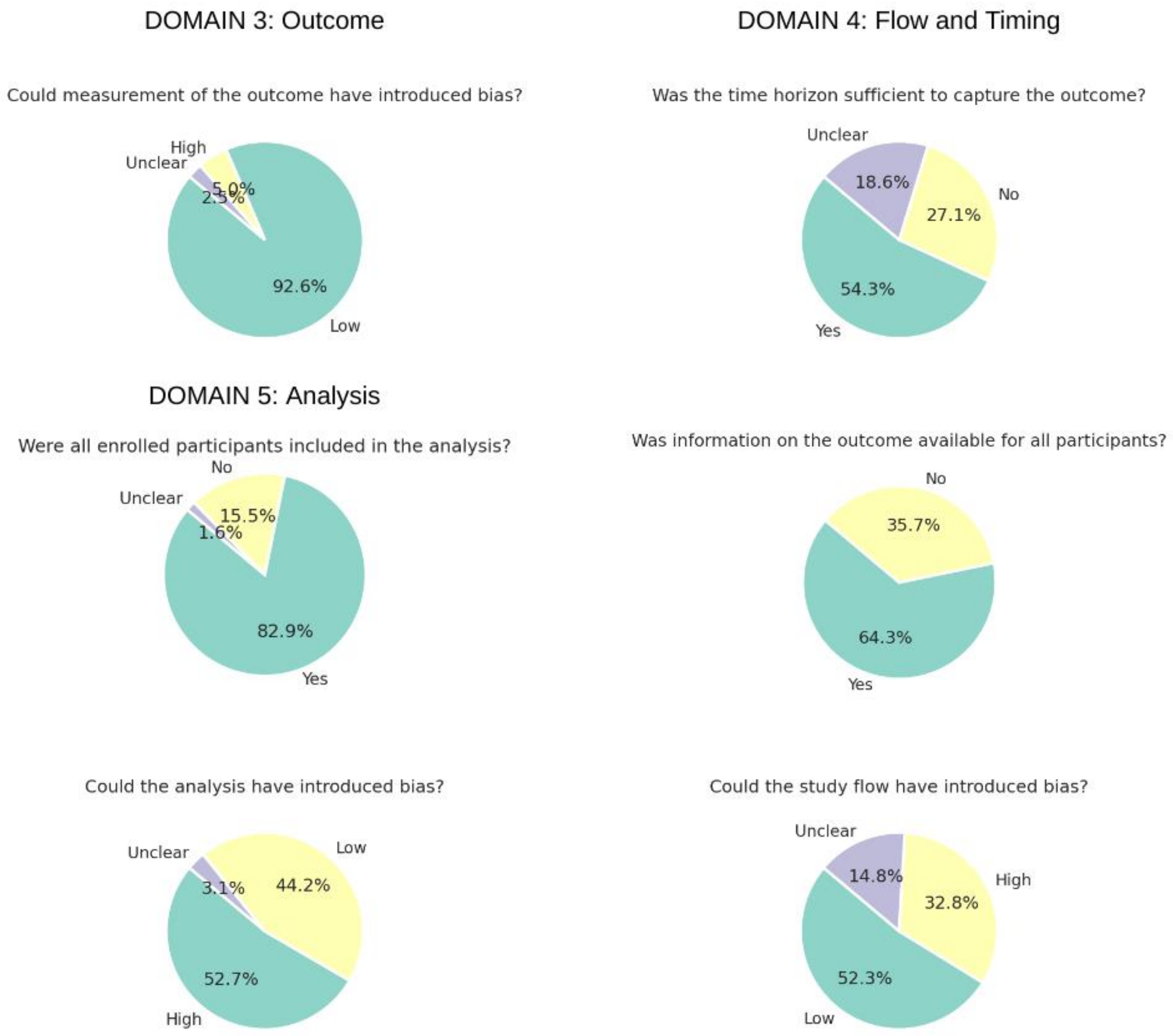

Figure S5. RoB assessment using the QUAPAS tool. Proportions of answers for signaling questions that provoked high RoB and the final RoB rating questions per domain.
